# Identification of New Antiseizure Medication Candidates in Preclinical Animal Studies

**DOI:** 10.3390/ijms241713143

**Published:** 2023-08-24

**Authors:** Chih-Sheng Yang, Man-Chun Wu, Ming-Chi Lai, Sheng-Nan Wu, Chin-Wei Huang

**Affiliations:** 1 Department of Neurology, Taichung Tzu Chi Hospital, Buddhist Tzu Chi Medical Foundation, Taichung City 42743, Taiwan; yashchun@gmail.com; 2 School of Post-Baccalaureate Chinese Medicine, Tzu Chi University, Hualien City 97004, Taiwan; 3 Department of Family Medicine and Preventive Medicine Center, Taichung Tzu Chi Hospital, Buddhist Tzu Chi Medical Foundation, Taichung City 42743, Taiwan; 4 Department of Pediatrics, Chi-Mei Medical Center, Tainan City 71004, Taiwan; vickylai621@gmail.com; 5 Department of Physiology, College of Medicine, National Cheng Kung University, Tainan City 70101, Taiwan; snwu@mail.ncku.edu.tw; 6 Institute of Basic Medical Sciences, College of Medicine, National Cheng Kung University, Tainan City 70101, Taiwan; 7 Department of Neurology, National Cheng Kung University Hospital, College of Medicine, National Cheng Kung University, Tainan City 70101, Taiwan

**Keywords:** animal models of seizures, maximal electroshock seizure model, kindling model, novel antiseizure candidates, drug-resistant epilepsy

## Abstract

Epilepsy is a multifactorial neurologic disease that often leads to many devastating disabilities and an enormous burden on the healthcare system. Until now, drug-resistant epilepsy has presented a major challenge for approximately 30% of the epileptic population. The present article summarizes the validated rodent models of seizures employed in pharmacological researches and comprehensively reviews updated advances of novel antiseizure candidates in the preclinical phase. Newly discovered compounds that demonstrate antiseizure efficacy in preclinical trials will be discussed in the review. It is inspiring that several candidates exert promising antiseizure activities in drug-resistant seizure models. The representative compounds consist of derivatives of hybrid compounds that integrate multiple approved antiseizure medications, novel positive allosteric modulators targeting subtype-selective γ-Aminobutyric acid type A receptors, and a derivative of cinnamamide. Although the precise molecular mechanism, pharmacokinetic properties, and safety are not yet fully clear in every novel antiseizure candidate, the adapted approaches to design novel antiseizure medications provide new insights to overcome drug-resistant epilepsy.

## 1. Introduction

Epilepsy is a common and disabling neurological disease characterized by recurrent and unpredictable seizures resulting from abnormally excessive synchronized discharges. It affects approximately 50 million people worldwide, accounting for a significant proportion of the socioeconomic burden, particularly in the economically disadvantaged countries [1,2]. Previous international epidemiological studies have reported a prevalence rate of active epilepsy ranging from 6.7 to 10 per 1000 persons [3,4]. The incidence rate of epilepsy in developed countries falls within the range of 40 to 70 per 100,000 person-years [5]. However, variations in statistical methodology, advancements in accurate diagnosis and classification, as well as differences in race and social class have contributed to disparities in research findings [6]. Epileptic seizures can have devastating consequences, including sudden unexpected death in epilepsy. Despite the availability of numerous antiseizure drugs on the market, achieving seizure freedom for epileptic patients remains a major challenge. Clinical studies have shown that over 30% of epilepsy patients are diagnosed with drug-resistant epilepsy (DRE) [7,8]. Several factors are associated with drug-resistant epilepsy, including symptomatic epilepsy, developmental retardation, abnormal neuroimaging or electroencephalography results, neuropsychiatric disorders, prolonged febrile seizures, and status epilepticus (SE) [9].

To solve the unmet demand of treating epilepsy successfully, recently expanding researches have been dedicated to exploring the underlying pathophysiological mechanisms of epileptogenesis and drug resistance. Currently, several representative hypotheses explaining the multifactorial causes of drug resistance have been reported, such as alteration of antiseizure targets that reduces the binding affinity of drugs [10] and overexpression of multidrug efflux transporter at the blood–brain barrier (BBB), restricting antiseizure medication (ASM) entry [11,12]; neuroinflammation contributing to BBB permeability dysfunction [13,14,15]; and aberrant neural network reorganization facilitating synchronization [16]. Furthermore, proactive investigations searching for novel antiseizure candidates are in progress. The researchers conducting these investigations attempt to modify chemical structures of existing ASMs or look for novel compounds targeting the relevant mechanism involved in pharmacoresistance and epileptogenesis.

Various animal models of seizures or epilepsy have been established for the screening and discovery of novel compounds to determine their efficacy and safety before entering clinical trials in humans. Rats and mice are the prevalent species of animal models to test the potential treatments. An animal model of seizures is an operative platform to estimate if those new compounds with antiseizure potential in in vitro studies have a promising antiseizure effect in in vivo studies. In addition, the animal models of seizures also provide information regarding pharmacokinetic and neurotoxicity of investigational compounds [17,18]. A highly validated animal model can predict the clinical response of investigational drugs on different types of seizures more closely. In general, the animal models for epilepsy researches are classified into two groups: models of epileptic seizures and models with chronic epilepsy [19]. The first group refers to electrically or chemically induced seizures in neurologically intact animals. For instance, the maximal electroshock seizure (MES) model and subcutaneous pentylenetetrazole (s.c. PTZ)-induced seizure model belong to the first group. The second group is characterized by spontaneous recurrent seizures (SRSs), such as inherent mutants or transgenic animals that are susceptible to SRS, as well as post-status epilepticus models with SRS. The selection of the most suitable animal models relies on the objectives of experiments. Most animal models utilized for discovery of compounds with antiseizure activities are models of epileptic seizures instead of models of chronic epilepsy due to time constraints and costs. For plenty of new antiseizure compound screening, MES and PTZ tests remain the two of the most time-efficient methods to determine the antiseizure potentials of the investigational compounds rapidly [20]. The kindling model has been the only chronic induced seizure model employed in new antiseizure discovery [21]. To overcome the DRE, there are increasing researches incorporating drug-resistant animal models as part of study protocols. On the contrary, post-SE models that display SRS and reproduce the characteristics of human temporal lobe epilepsy (TLE) generally serve for purposes of new antiepileptogenic or disease-modifying therapy development [22,23]. 

With an increasing number of compounds showing potential as antiseizure agents, there is a concern regarding whether these novel candidates can offer more benefits compared to the established ASMs. The investigation of novel antiseizure candidates in animal models of seizures is also essential to understand whether their antiseizure effects can address different seizure types and explore innovative mechanisms of action. In light of the context described above, this review aims to summarize the validated rodent models of seizures commonly utilized in the discovery of novel ASMs. Additionally, it will discuss the latest research within the last decade that focuses on identifying new antiseizure candidates during the preclinical stage using animal models of seizures, as well as the proposed mechanisms by which they suppress epileptic seizures.

## 2. Scope of Review

**Figure 1:**
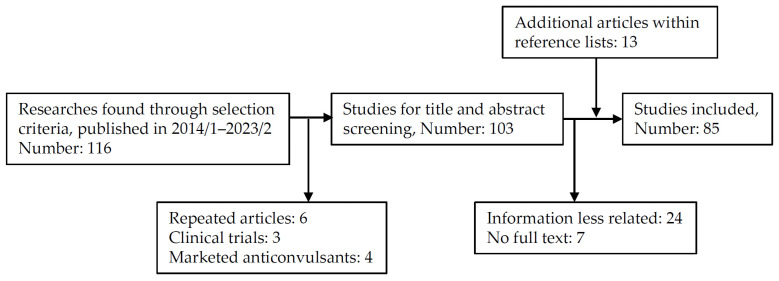
Flow diagram of database search.

In this literature review, we used the following academic databases: PubMed and MEDLINE. The inclusion criteria of articles included peer-reviewed articles consisting of original articles, case reports, reviews, systemic reviews, and meta-analyses. We aimed to review the novel compounds that exert promising antiseizure activities in animal models of seizures or epilepsy and excluded the in vitro cellular or hippocampal slice studies. Those studies describing new ASMs under the clinic trials or marketed ASMs were also excluded. The search keywords were ((new OR novel OR innovative) AND (antiepileptic candidate OR anticonvulsive candidate OR antiseizure candidate)) AND (animal model). The researches published between 1 January 2014 and 28 February 2023 and written in English were used. After initial inspection of titles and abstracts, the full texts of articles considered for further inclusion were thoroughly screened (Figure 1). We excluded the following articles whose full texts did not involve new antiseizure agents or animal models of seizures. 

## 3. Animal Models Used for ASM Screening and Discovery 

### 3.1. Maximal Electroshock Seizure (MES) Model 

The MES model is one of the most commonly employed animal models for new antiseizure drug discovery. Generalized tonic–clonic (GTC) seizures are elicited through ear clip stimulation of a short (0.2 s), suprathreshold electric current in healthy experimental mice (50 mA) or rats (150 mA). Induced seizures manifest as three stages: tonic hindlimb flexion lasting for a few seconds; generalized tonic extension lasting for 10–15 s; and the recovery stage. The results of the MES test are interpreted as positive when the experimental mice or rats present with tonic hindlimb extension posture sustained for 3 s within 10 s after MES stimulation [24,25]. Median effective dose (ED_50_) represents a median effective dose at which the investigational compound can protect 50% of mice from MES-induced seizures. The log-probit analysis is a simplified method used to determine the ED_50_ value [26]. The MES model represents a predictive model of generalized tonic–clonic seizures [27]. Most ASMs that display antiseizure activities in the rodent MES model have been proven to be effective in clinical patients with generalized tonic–clonic seizures [28]. Moreover, some ASMs that fail to counter MES-induced seizures are ineffective in treatment of human GTC seizures, including ethosuximide, tiagabine, and vigabatrin [29]. Notably, previous research has highlighted the unique characteristics of levetiracetam in comparison to other ASMs. Despite its lack of effectiveness against MES-induced seizures, levetiracetam has been clinically approved for treating primary GTC seizures [30,31]. The predictive value of ASM activities against focal seizures has also been extensively studied using the MES model. Interestingly, levetiracetam, tiagabine, and vigabatrin, all of which do not demonstrate antiseizure/anticonvulsant effects in MES-induced seizures, have shown promising results in the clinical treatment of focal epilepsy [32]. This disparity between preclinical animal studies and clinical trials suggests that the MES model may not be a perfect representation of focal seizures.

Given the evidence mentioned above, the MES model still serves as a time-efficient screening model for the discovery of novel antiseizure drugs. However, in cases where investigational compounds fail to show effectiveness in the MES model, it is crucial to consider other animal models, such as the s.c. PTZ model or the kindling model, to explore potential antiseizure benefits with a novel mechanism.

### 3.2. Subcutaneous Pentylenetetrazole-Induced Seizure Model 

The subcutaneous administration of PTZ at a convulsive dose of 85 mg/kg (mice) or 70 mg/kg (rats) induces clonic seizures [33]. After PTZ injection, the experimental animals are observed for a period of 30 min to monitor seizure occurrence. The clonic seizure activities manifest as clonus movement of the whole body lasting for over 5 s, accompanied by loss of righting reflex. The animals are pretreated with investigational compounds, and the absence of consequent clonic spasms after PTZ induction indicates antiseizure abilities of investigational compounds. The antiseizure activity of each compound is measured as the ED_50_ value [26]. The s.c. PTZ test has been considered as a predictive of antiseizure activities countering generalized nonconvulsive myoclonic and generalized spike-wave seizures [29]. As reported by earlier studies, trimethadione that was effective against PTZ-induced seizures demonstrated therapeutic benefits in the treatment of absence seizures [34,35]. Moreover, ethosuximide and succinimide were also identified by s.c. PTZ tests, showing evidence of treating generalized absence epilepsy [36]. Nevertheless, subsequent researches provided a contrary point of view. Gabapentin and tiagabine exerted effectiveness against s.c. PTZ-induced seizures but aggravated generalized spike-wave seizures clinically [37,38]. Furthermore, some ASMs that failed in the s.c. PTZ test showed protection in patients with absence or myoclonic epilepsy, such as levetiracetam and topiramate [39]. As a result, the preclinical studies using an s.c. PTZ-induced seizure model cannot extensively predict the antiseizure effects of investigational compounds on generalized absence epilepsy or myoclonic epilepsy. A more precise prediction of the antiseizure activities on these generalized nonconvulsive seizures necessitates further examinations with other genetic mutant models including WAG/Rij and genetic absence epilepsy rats from Strasbourg (GAERS) models that exhibit spontaneous spike-wave discharges [40]. 

### 3.3. Kindling Model 

In contrast to the MES model and s.c. PTZ model that elicit acute seizure in healthy rodents, the kindling model represents a chronic model in which repeated seizures are induced via electrical or chemical stimuli to achieve enduring neural network hyperexcitability [41]. The kindling model remains the only chronic model accepted by most antiseizure development programs [42]. In amygdala- or hippocampal-kindled rodent models, the depth electrodes are implanted into the limbic brain region first. The rats receive repeated suprathreshold electric stimuli of 200 µA for 10 s every 30 min for 6 h on alternative days until fully kindled. After the experimental rats reach a fully kindled state, the investigational compounds are administered as study protocols. The rats are challenged with electric kindling stimulus later. The rats are considered as protected if their behavioral seizure score is equal to three or less, based on the Racine scale system [43]. For chemical kindling induction, such as PTZ, a selective antagonist of γ-Aminobutyric acid type A (GABA_A_) receptor, the experimental animals are repetitively injected with PTZ at a subconvulsive dose of 40 mg/kg on every other day for a number of days [44]. After each PTZ injection, the experimental animals are isolated and observed for 30 min to assess the consequent seizure score [45]. The antiseizure effect of investigational compounds is further measured in fully kindled animals. 

Through increasing seizure susceptibility and neural network alteration in response to repetitive stimulation of the limbic system with an initially subconvulsive current for weeks, the kindled rodent models gradually develop many characteristics that resemble human TLE, including chronic seizure activity, histologically neuronal degeneration, and cognitive deficits [46,47,48,49]. Given the similarity with human TLE, the kindling model plays a valuable role in predicting antiseizure activities against focal epilepsy. The endpoint of measuring the effect of investigational ASMs relies on the consequent evoked seizure scores of experimental animals. As shown by earlier studies, the amygdala-kindled model displayed its predictive validity by accurately exploring the potent antiseizure potential of levetiracetam, one of the most commonly used ASMs to treat human focal epilepsy currently [30]. Furthermore, other ASMs that can block seizures in kindling rodent models have been proven to be effective in human focal epilepsy [32]. It is particularly worth mentioning that employing a battery of additional models, like the kindling model, is important to avoid missing a potential antiseizure, as there is no promising effect documented in initial screening models such as the MES or s.c. PTZ model. 

### 3.4. Animal Models for Pharmacoresistant Epilepsy

#### 3.4.1. 6 Hz Psychomotor Seizure Model 

The 6 Hz psychomotor seizure model is an alternative electroshock method involving the application of low-frequency (6 Hz), long-duration (3 s) electric stimulation [50]. Seizures are induced in neurologically intact mice or rats through corneal stimulation, achieved using a constant-current device that delivers 6 Hz, monopolar, rectangular pulses for 3 s after local anesthesia [51]. Following the electric stimulation, the mice are observed for any subsequent behavioral changes.

During the seizures, the mice display immobility, forelimb clonus, twitching of the vibrissae, a stiffened tail, and rearing behavior. However, it is important to note that these experimental mice are considered “protected cases” as they return to their normal behavior patterns within 10 s after the electric stimulation ends [52]. The ED_50_ value is determined by the log-probit method. It has been reported that most marketed ASMs lacked effectiveness against 6 Hz seizures when 44 mA current was applied, except valproate, levetiracetam, brivaracetam, and carisbamate [53]. Consequently, the 6 Hz seizure model provide a useful and time-efficient screening approach to identify potential ASMs against DRE. 

#### 3.4.2. Lamotrigine (LTG)-Resistant Amygdala-Kindled Seizure Model 

As previously mentioned, in the kindling process in amygdala-kindled models, the experimental rats receive repetitive electrical stimuli with a suprathreshold current (200 µA) until the rat displays fully kindled behavior. Notably, during the period of kindling acquisition, intraperitoneal lamotrigine (5 mg/kg, i.p.) is administered one hour before each amygdala stimulation, diminishing the subsequent response to LTG in fully kindled animals [54]. One week after the rats achieve the fully kindled state, they receive an LTG challenge with a higher dose (15 mg/kg, i.p.) to confirm the LTG-resistance seizure model. LTG-resistant kindled animals have been regarded as models of DRE, in which carbamazepine, phenytoin, and topiramate have been confirmed ineffective to the model [55]. The absence of efficacy of some ASMs acting on the same mechanism with LTG might be attributed, in part, to cross-tolerance. Nonetheless, the role of cross-tolerance in human DRE remains not fully clear [56]. 

#### 3.4.3. Post-Status Epilepticus Seizure Model 

TLE with epileptogenic foci in the limbic system remains one of the most common forms of refractory focal epilepsy in the adult population [57,58]. The post-SE model reproduces clinical characteristics resembling human TLE, including precipitating brain insults, a latent period between the initial insults, and the first spontaneous seizure and chronic SRS [59]. Moreover, there are TLE-mimic hippocampal neuronal damages shown in the histopathology of the post-SE model [60]. The initial status epilepticus is induced by either chemical stimulation (pilocarpine, kainic acid) or electrical stimulation (basolateral amygdala, perforanth). As reported by an earlier study, in a pilocarpine-induced SE model, prolonged administration of levetiracetam demonstrated interindividual variation in drug response [61]. Similarly, prolonged use of phenobarbital at maximal tolerated doses also resulted in two distinct subgroups—responders and nonresponders—in an electrically induced SE model [62]. The majority of experimental rats with phenobarbital-resistant seizures have been refractory to other ASMs, such as phenytoin, suggesting nonresponders to an initial ASM are likely to be resistant to a second ASM [63]. By selecting nonresponders of multiple traditional ASMs in kindling models or post-SE models, advancing pharmacoresistant animal models are established for further researches of underlying mechanisms contributing to antiseizure resistance. 

### 3.5. Evaluation of Antiseizure Potentials of Novel Compounds in Preclinical Animal Studies 

**Figure 2:**
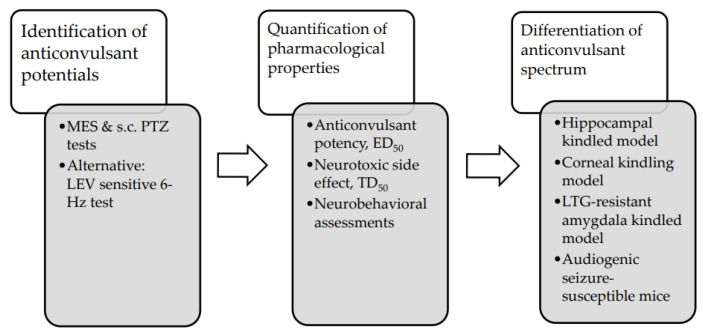
Schematic flowsheet of novel ASM identification and development, proposed by The Epilepsy Therapy Screening Program, formerly known as ASM Screening Program. Adapted from [19]. 2011, Löscher and Wilcox. Abbreviations: ED_50_ = median effective dose, LEV = Levetiracetam, LTG = Lamotrigine, MES = maximal electroshock seizure, PTZ = pentylenetetrazole, TD_50_ = median toxic dose.

As shown in the diagram of the ASM screening program (Figure 2), an investigational compound is initially evaluated for its antiseizure potential in the MES and s.c. PTZ tests responsible for rapid screening and identification of potential treatments. If the compound fails to suppress the seizure induced by the MES or s.c. PTZ test, a levetiracetam-sensitive 6 Hz test is subsequently utilized [19]. Those compounds exerting antiseizure activities in one or more of these three screening models are quantitated for their pharmacological properties and neurotoxicity. Additional seizure models, including hippocampal/amygdala-kindled models, are warranted to determine and differentiate the antiseizure spectrum of the novel antiseizure candidates with promising efficacy in screening models and minimal neurotoxicity.

### 3.6. The Potential Benefits and Limitations of Animal Models of Induced Seizures 

As mentioned earlier, acute seizure models for novel antiseizure medication screening are time-efficient and high-throughput tools to discover new drugs in the preclinical phase. Easy implementation, low cost, and simplicity are their advantages. They also play an essential role in evaluating pharmacokinetic properties and adverse effects of investigational compounds, such as neurotoxicity and hepatotoxicity before clinical trials. Furthermore, it should be mentioned that animal models are unbiased with respect to assumptions about the specific targets the drugs act on. Nevertheless, animal models that exhibit acute seizures after electrical or chemical induction have limited mechanistic relevance to human epilepsies. Moreover, it should be concerning that variation in the seizure threshold and the potency of drugs potentially complicate data interpretation when different species are compared in the MES seizure model and 6 Hz psychomotor seizure model [53]. Kindling models recapitulate the epileptogenesis process and have high predictive validity for focal seizures [21]. However, the limitations of a time-consuming, highly technique-dependent procedure make them unsuitable for high-throughput screening. Herein, we summarized the important, representative characteristics of the aforementioned seizure models in Table 1.

## 4. New Antiseizure Candidates

### 4.1. N-benzyl-2-(2,5-dioxopyrrolidin-1-yl)propanamide (AS-1)

N-benzyl-(2,5-dioxopyrrolidin-1-yl)propanamide (AS-1) is synthesized as an integrated hybrid compound which combines structural fragments of three marketed ASMs—ethosuximide (ETX, pyrrolidine-2,5-dione derivative), levetiracetam (LEV, pyrrolidin-2-one derivative), and lacosamide (LCS, classified as functionalized amino acid)—through molecular hybridization [64,65]. In preclinical researches for initial ASM discovery, AS-1 exhibited its potent antiseizure activities in a number of validated acute seizure models, such as the MES test, s.c. PTZ test, and the 6 Hz (32 mA) test, in mice [51,66]. After confirmation of antiseizure potentials in screening models such as the MES and s.c. PTZ tests, a battery of other seizure models was employed to explore the spectrum of antiseizure activities. As shown in a recent comprehensive research, AS-1 significantly suppressed the progression of kindling induced by repeated PTZ injection in mice. Furthermore, in a 6 Hz (44 mA) model that represents a DRE model, AS-1 also revealed a protective effect countering induced seizure. Isobolographic analysis disclosed synergistic interaction of AS-1 and valproic acid in PTZ-induced clonic seizures, suggesting probable additional benefits provided by combination therapy. Derived from AS-1, R-enantiomer of AS-1 (R)-7[(R)-AS-1] also displays wide-spectrum antiseizure activities across several of acute seizure models in mice. The mechanism that (R)-7[(R)-AS-1] acts on may arise from modulation of the excitatory amino acid transporter-2, one of the glutamate transporters [67]. In in vitro studies to estimate pharmacokinetics and drug safety, AS-1 did not affect the function of most cytochrome P450 (CYP) enzymes (CYP3A4/CYP2D6), and only moderate inhibition of CYP2C9 at a higher concentration of 10 µM was observed. There was no hepatotoxicity in the HepG2 cell line at the concentration of 10 µM. However, statistical decline of HepG2 cell viability was reported at the highest dose, 100 µM [68]. It is well known that phenytoin (PHT) and valproic acid (VPA) are metabolized by both CYP2C9 and CYP2C19. Therefore, the drug–drug interaction between AS-1 and PHT/VPA is worth special attention in further clinical trials. Taken together, AS-1 has been documented as a new broad-spectrum antiseizure candidate with apposite safety profiles. Nonetheless, in vitro functional/binding assays failed to determine the target upon which AS-1 acts [66]. More investigations to explore the definite mechanism of antiseizure activity are warranted. 

### 4.2. 2-(2,5-dioxopyrrolidin-1-yl) propanamide derivative (C-11)

Similar to AS-1, the same research team developed a series of multifunctional ASMs with hybrid structures integrating fragments of ethosuximide, levetiracetam, and lacosamide [69,70,71]. C-11 (former name KA-11) is recognized as a new hybrid compound derived from 2-(2,5-dioxopyrrolidin-1-yl) propanamide [72]. As shown in previous quantitative studies, C-11 demonstrated promising, broad-spectrum antiseizure activities in several preclinical animal models including MES, s.c. PTZ, and 6 Hz (32 mA) psychomotor seizure models [73]. In a PTZ-induced chronic kindling model, repeated C-11 administration attenuated kindling progression in experimental mice. In addition, the compound did not give rise to acute perturbation of motor coordination even at very high doses (median toxic dose, TD_50_ > 1500/kg), indicating its acceptable safety property. Regarding hepatotoxicity, a slight decrease of HepG2 cells’ viability was reported only at the highest dose, 100 µM. Based on the electrophysiological experiments by the patch-clamp technique, one of the antiseizure mechanisms stemmed from inhibition of voltage-gated sodium channels [74]. In addition to antiseizure potentials, it is worth mentioning that the learning and memory function as well as neurogenesis process of experimental mice had not been affected after chronic C-11 administration [75]. In general, C-11, a new synthesized hybrid, has proved to be a wide-spectrum antiseizure candidate with negligible neurotoxicity, providing a new insight into ASM discovery in the future. 

### 4.3. 2-(2′-fluoro-[1,1′-biphenyl]-2-yl)acetamide (DSP-0565)

2-(2′-fluoro-[1,1′-biphenyl]-2-yl)acetamide (DSP-0565) is a novel compound that exhibits antiseizure activities in MES, s.c. PTZ, and 6 Hz seizure models as well as acceptable neurotoxicity in the rotaroad test. Moreover, in an amygdala kindling model, DSP-0565 significantly reduced the after-discharge duration (ADD) and seizure stages as well as improving after-discharge threshold (ADT) [76]. As mentioned above, the amygdala-kindled model has been employed to predict the antiseizure potential against focal seizure in clinical studies. As a result, DSP-0565 has been identified to be a broad-spectrum antiseizure candidate. The investigators initially found that 2′-fluoro-N-methyl-[1,1′-biphenyl]-2-sulfonamide showed potent ASM activities in a number of seizure screening models, but its reactive metabolite led to the risk of unfavorable idiosyncratic toxicity [77]. To diminish reactive metabolite production, they identified an alternative polar group and optimized N-methylsulfonamide moiety in the previous lead compound. Of the resultant ortho-aryl-phenyl acetamide derivatives, DSP-0565 demonstrated the better pharmacokinetic characteristics and safety margin compared to ortho-aryl-phenyl sulfonamide derivatives [76]. The exact mechanism of antiseizure action of DSP-0565 remains undetermined at present, and more investigations are required. 

### 4.4. S(+)-(2E)-N-(2-hydroxypropyl)-3-phenylprop-2-enamide (KM-568)

S(+)-(2E)-N-(2-hydroxypropyl)-3-phenylprop-2-enamide (KM-568) is a cinnamamide derivative. Cinnamamide, a privileged scaffold, derives from cinnamic acid that is a kind of organic acid existing in natural plants. As shown in earlier studies, some cinnamamide derivatives demonstrated widely therapeutic efficacy in numerous neurological disorders including epilepsy, depression, neuropathic pain, and sleep disorders. [78,79]. The compound KM-568 protected experimental animals from seizure in several acute induced seizure models including the MES test, 6 Hz (32 and 44 mA) psychomotor seizures, and seizures induced by chemoconvulsants. Moreover, in a genetically modified Frings audiogenic seizure-susceptible mouse model of epilepsy, corneal-kindled mouse models, a hippocampal-kindled rat model, and a lamotrigine-resistant amygdala-kindled model, KM-568 has proved to be effective as well. The dose–response relationship was obtained. In the light of the aforementioned results, it is worth our attention that KM-568 probably plays a beneficial role in treating drug-resistant epilepsy. Cytotoxicity studies using HepG2 and H9c2 cell lines reported the safety of the compound KM-568 in a concentration up to 100 µM. To explore the mechanism of action, three radioligand binding assays targeting GABA receptor, α-amino-3-hydroxy-5-methyl-4-isoxazolepropionic acid (AMPA) receptor, and N-methyl-D-aspartate (NMDA) receptor were applied, but there was no significant interaction observed in these tested targets [33]. Further investigations are ongoing. 

### 4.5. 5-(3-chlorophenyl)-4-hexyl-2,4-dihydro-3H-1,2,4-triazole-3-thione (TP-315)

In recent published studies, it has been reported that some 1,2,4-triazole-3-thione derivatives displayed antiseizure activities against MES-induced seizures and 6 Hz (32 mA) psychomotor seizures [80,81,82]. 5-(3-chlorophenyl)-4-hexyl-2,4-dihydro-3H-1,2,4-triazole-3-thione (TP-315) was identified as one of the 1,2,4-triazole-3-thione derivatives with potent antiseizure potentials and gained more interest in its mechanism and long-term effect on living organisms. The interactions between TP-315 and different targets, such as GABA_A_ receptors, voltage-gated sodium channels (VGSCs), and nicotinic acetylcholine receptors (nAChRs), were approached. Radioligand binding assays confirmed that TP-315 was a very effective sodium channel blocker. There was neither modulation on GABA_A_ receptor chloride currents nor interaction with nAChRs [83]. In addition to promising antiseizure potentials, TP-315 possesses acceptable safety properties. As reported by a recent study, TP-315 slightly affects the viability of HepG2 cells at the highest concentration of 100 µM. Moreover, in living organisms, there was neither alteration of hepatic and renal function nor change of hematologic parameters after long-term administration of TP-315. At a concentration similar to the concentration measured in the tested mice, the activities of the enzymes CYP2B6, CYP2D6, CYP3A4, and CYP3A5 were not significantly inhibited by TP-315, although CYP2C19 was [84]. Potential interaction with some marketed ASMs that are substrates of CYP2C19, such as diazepam, phenytoin, and valproic acid, deserves more investigations. 

### 4.6. Neuroactive Steroid (SGE-516)

SGE-516 is a synthetic neuroactive steroid designed with favorable pharmacokinetic properties for chronic oral dosing. In earlier studies, neuroactive steroids have been documented to modulate both synaptic and extrasynaptic GABA_A_ receptors and enhance GABA-mediated chloride currents [85,86]. It is worth mentioning that SGE-516 is identified as a positive allosteric modulator of both gamma- and delta-containing GABA_A_ receptors. Compared to benzodiazepines, which selectively act on gamma subunit-containing GABA_A_ receptors, the neuroactive steroid SGE-516 possesses broader GABA_A_ receptor activities [87,88]. In preclinical mice studies, SGE-516 had proved to suppress acute induced seizures successfully in PTZ and 6 Hz psychomotor seizure models in a dose–response manner. Furthermore, SGE-516 also exhibited its potent antiseizure activities in a corneal-kindled model and lithium–pilocarpine model [89,90]. The broad-spectrum antiseizure properties and novel mechanism of SGE-516 potentiating α1β2γ2 and α4β3δ subunit-containing GABAergic transmission may provide inspiration in the treatment of drug-resistant epilepsy. 

### 4.7. Methylsulfonyl Phenyl Derivative (MTL-1)

MTL-1, a methylsulfonyl phenyl derivative, is developed and synthesized as a potent and selective Cyclooxygenase-2 (COX-2) inhibitor through combining the properties of methylsulfonyl benzene, urea, and thiourea functional groups [91]. The potency and selectivity of MTL-1 on inhibition of COX-2 as well as its safety has been established by serial molecular biological studies [92,93]. In preclinical animal studies of new ASM discovery, MTL-1 effectively protected experimental mice against s.c. PTZ-induced seizures. Moreover, in a chronic PTZ-induced kindled rat model, significant reduction of evoked seizure severity was obtained by administration of MTL-1. Beyond the antiseizure potentials, MTL-1 showed benefits in social and novel object recognition in PTZ-kindled rats [94]. Rapidly expanding researches supported the conclusion that the neuroinflammation process comprising activation of glial cells, release of inflammatory molecules, and blood–brain barrier (BBB) destruction plays an essential role in epileptogenesis after initial brain insults [14,95]. Overexpression of COX-2 was discovered in the hippocampal pyramidal cells of epileptic rats [96]. Thus, anti-inflammatory molecules with antiseizure activities as new antiseizure candidates deserve more attention in further clinical studies. 

### 4.8. Sebacic Acid

Among these hypotheses explaining why some epilepsies evolve into drug-resistant epilepsy, the transporter hypothesis that refers to overexpression of a multidrug efflux transporter, such as P-glycoprotein (P-gp), causing restricted penetration of ASMs across the BBB, attracts a lot of interest. Overcoming the upregulation of P-gp is considered as feasible treatment in drug-resistant epilepsy. Sebacic acid (SA) is a straight-chain, ten-carbon aliphatic dicarboxylic acid normally made from castor oil [97]. In a recently published study, it was reported that SA demonstrated antiseizure activities in MES- and s.c. PTZ-induced seizure models. In addition, sebacic acid reverses the overexpression of P-gp in the hippocampus, striatum, and cerebral cortex in experimental mice [98]. However, sebacic acid is mainly synthesized for industrial or laboratory use and can cause irritation of eye, skin, and respiratory tract as an unwanted effect. The pharmacokinetic profiles, hepatotoxicity, as well as the safety after chronic administration of this compound are unknown. More detailed preclinical safety studies are mandatory. 

### 4.9. (S)-3-amino-4-(difluoromethylenyl)-cyclopent-1-ene-1-carboxylic Acid (OV329)

γ-Aminobutyric acid (GABA) has been well known as the most important inhibitory neurotransmitter in the mature brain. Enhancing GABAergic transmission by raising GABA concentration has been employed to control seizures by some ASMs such as vigabatrin and tiagabine [99]. Vigabatrin, an inhibitor of GABA aminotransferase (GABA-AT), is approved as an add-on therapy for focal epilepsies in adults and infantile spasms in pediatric patients [100]. Nevertheless, the disadvantages of vigabatrin, including relatively poor potency and the severe adverse effect of retinal damage with prolonged use, lead to its limited utility [101]. Given the aforementioned drawbacks of vigabatrin, OV329 has been a refined, more potent inactivator of GABA-AT than vigabatrin [102]. In preclinical animal studies, OV329 significantly increased the threshold of intravenous (i.v.) PTZ-induced acute seizures. Moreover, in amygdala-kindled rats as a predictive model of human TLE, OV329 increased the ADT and reduced the ADD as well as evoked seizure severity in a dose–response manner [103]. Notably, OV329 exhibited 30-fold greater antiseizure potency and better tolerability on i.v. PTZ-induced myoclonic seizures compared to previous investigation of vigabatrin [104]. OV329, a novel GABA-AT inactivator, shows an admirable antiseizure property in in vivo studies. Further examinations to evaluate the toxic effect on the nervous system and hepatocellular cells are required. 

### 4.10. 5-(8-ethynyl-6-(pyridin-2-yl)-4H-benzo[f] imidazo [1,5-a][1,4]diazepin-3-yl)oxazole (KRM-II-81)

5-(8-ethynyl-6-(pyridin-2-yl)-4H-benzo[f]-imidazo [1,5-a][1,4]diazepin-3-yl)oxazole, KRM-II-81 acts as a positive allosteric modulator (PAM) on the α2/3-containing GABA_A_ receptors exclusively [105]. The advantages of a selective modulator of α2/3-containing GABA_A_ receptor include reduced risk of sedation, motor impairment, and abuse, compared to nonselective PAM, such as diazepam, one of the most employed benzodiazepines to suppress acute seizure currently [106,107,108]. In acute seizure models for ASM screening, it was reported that KRM-II-81 was effective in suppressing MES-induced seizures in mice, and s.c. PTZ evoked convulsions in rats, respectively. The broad antiseizure activities of KRM-II-81 have also been identified in an amygdala-kindled rat model that was regarded as a predictive model of human TLE [109]. It was worth our attention that KRM-II-81 demonstrated its commendable efficacy in DRE models, including a 6 Hz (44 mA) seizure model in mice, lamotrigine-resistant amygdala kindled seizure model, and kainate-induced SE models [105,109]. Based on the unique mechanism and promising antiseizure potentials in DRE rodent models, KRM-II-81 may represent a broader-spectrum antiseizure candidate with less adverse effects compared to currently available ASMs such as diazepam [110].

## 5. The Potentials of Novel ASM Discovery to Surmount Drug Resistance

Although notably expanding ASMs had been approved over the past decades, the percentage of drug-resistant epilepsy did not significantly decline [111]. In order to overcome the clinically unsatisfactory dilemma, many researchers in pharmaceutical institutes adopted strategies and took the following approaches for new ASM development: structural modification of existing marketed ASMs, exploring antiseizure candidates acting on innovative mechanisms, and nonmechanism-based screening in animal models of seizures [112].

We summarized the antiseizure spectrum and postulated mechanism of new antiseizure candidates described earlier in Table 2. Among these novel antiseizure candidates, C-11 and TP-315 have a mechanism of action similar with previous marketed ASMs that inhibit VGSC. Although MTL-1 and sebacic acid target innovative mechanisms, like COX-2 inhibition and attenuating P-gp overexpression, respectively, which have shown preliminary ASM effects in screening models, the response to an amygdala-kindled model or DRE model was unclear.

Reviewing aforementioned ASM candidates with convincing efficacy on DRE models, AS-1 and C-11 are different derivatives of hybrid compounds integrating chemical structures of approved efficacious ASMs (ethosuximide, levetiracetam, and lacosamide) [64,65,69]. They exhibit broad-spectrum antiseizure activities against DRE models. Furthermore, they could possess the advantages of monotherapy. Different from diazepam, a nonselective GABA_A_ PAM, SGE-516 and KRM-II-81 have been identified as novel PAMs on subtype-selective GABA_A_ receptors, demonstrating promising efficacy on DRE models [89,90,109]. The specific subtypes of GABA_A_ receptors represent attractive targets of novel antiseizure candidates in term of their potency and better tolerability. KM-568 refers to a derivative of cinnamamide isolated from natural plants and has proven to be an effective drug in various DRE models [33]. However, the exact mechanism of KM-568 is undetermined so far. On the other hand, many researches have focused on the targets involved with epileptogenesis and pharmacoresistance, so some chemical compounds that can attenuate neuroinflammation or P-glycoprotein overexpression have been screened in acute and chronic evoked seizure animal models for determining their antiseizure potentials, such as sebacic acid and MTL-1. More investigations employing a battery of drug-resistant models to determine their antiseizure spectrum are warranted in the future.

## 6. Conclusions

Validated animal models of seizures have played a crucial role in discovering new antiseizure candidates, determining the range of effects of investigational compounds as ASMs, and evaluating drug safety. The selection of specific animal models depends on the objectives of the experiments. In recent years, several promising novel ASM candidates have emerged, including hybrid compounds that combine multiple existing ASMs, novel positive allosteric modulators (PAMs) targeting subtype-selective GABA_A_ receptors, and a derivative of cinnamamide. Unlike most conventional ASMs that modulate ion channels, there has been a growing interest in innovative agents targeting the underlying mechanisms of pharmacoresistance. These novel ASM candidates have drawn attention due to their potential to overcome drug resistance. However, before proceeding to clinical trials, further investigations are necessary to assess their pharmacokinetic properties and safety margins. Thorough evaluation of these compounds, which have demonstrated effectiveness in preclinical animal models, is essential.

## Figures and Tables

**Table 1:** Characteristics of acute/chronic seizure rodent models employed in ASM discovery.

Model	Induction Methods	Clinical Relevance	Advantages	Disadvantages	Reference
Acute seizure model					
Maximal electroshock seizure model	Corneal or auricular stimulation with suprathreshold electric current	Generalized tonic–clonic seizures	High throughput and time-efficient for ASM screening Reproducibility Low cost Easy implementation	Lack of predictive validity for some ASMs, such as levetiracetam, tiagabine, and vigabatrin Species difference Limited mechanistic insights	[24,25,27,28,30,31]
Subcutaneous pentylenetetrazole-induced seizure model	Subcutaneous pentylenetetrazole injection	Generalized myoclonus and spike-wave seizures	Inexpensive and time-efficient for ASM screening Simplicity Standardization of induction Rapid onset of seizures	Low predictive validity in ASM effectiveness Lack of clinical relevance for all seizure types Limited mechanistic insights	[29,33,37,38,39]
Chronic seizure model					
Kindling model	Repeated seizure induction via electrical/chemical stimuli	Focal seizures	High predictive validity for focal seizures Recapitulation of epileptogenesis Etiologically relevant model Translational relevance	Expensive and time-consuming Individual variability Technique demand Complexity of data interpretation	[32,41,42,44,46,47,48,49]
**Models for Pharmacoresistant Epilepsy**	**Induction Methods**	**Clinical Relevance**	**Advantages**	**Disadvantages**	**Reference**
6 Hz psychomotor seizure model	6 Hz, 3 s currents with intensity of 22 mA to 44 mA delivered to anesthetized corneas	Focal psychomotor seizures Pharmacoresistant seizures evoked by 44 mA	Differentiation of new ASMs against drug-resistant seizures Rapid and inexpensive test Simple stimulation protocol Reproducibility	Species difference Limited clinical relevance	[51,52,53]
Lamotrigine-resistant amygdala-kindled seizure model	Treatment of a low dose of LTG prior to each stimulation during kindling acquisition	Drug-resistant chronic focal seizures	Differentiation of novel ASMs against pharmacoresistant seizures Investigating mechanisms of drug resistance	The tolerability of some ASMs differs compared to LTG-naive mice Limited clinical relevance Complex drug resistance mechanism	[54,55]
Post-status epilepticus seizure model	Status epilepticus evoked by either electrical stimulation or chemical stimulation	Human mesial temporal lobe epilepsy	Recapitulation of human TLE High translational relevance with SRS Exploration of pathophysiology of epileptogenesis	High mortality Variable intensity of evoked status epilepticus and consequential seizures Labor intensive and time-consuming Interpretation of SRS	[59,60,62,63]

Abbreviations: ASM = antiseizure medication, LTG = Lamotrigine, SRS = spontaneous recurrent seizure, TLE = temporal lobe epilepsy.

**Table 2:** The antiseizure spectrum and postulated mechanism of new antiseizure candidates in preclinical animal studies.

Drug Name	Chemical Formula	Antiseizure Effects on Models of						Postulated Antiseizure Mechanism		Reference
		Maximal Electroshock Seizure	s.c. PTZ-Induced Seizure	Amygdala- or Hippocampal-Kindled Rat	PTZ-Induced Kindling	6 Hz Psychomotor Seizure	LTG-Resistant Amygdala-Kindled Seizure	Miscellaneous Batteryof Seizures		
AS-1	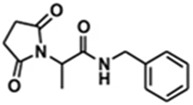	+	+	ND	+	+	ND	-	Unclear	[51,64,65,66,67,68]
C-11	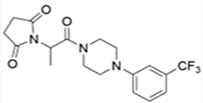	+	+	ND	+	+	ND	-	Inhibition of VGSC	[72,73,74]
DSP-0565	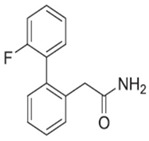	+	+	+	ND	+	ND	-	Unclear	[76,77]
KM-568	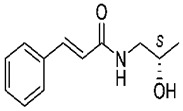	+	+	+	ND	+	+	Corneal kindling	Unclear	[33]
TP-315	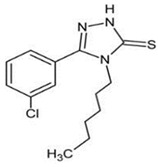	+	ND	ND	ND	+	ND	-	Inhibition of VGSC	[80,81,82,83]
SGE-516	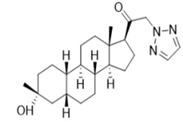	ND	+	ND	ND	+	ND	Corneal kindling, Li-Pilo-evoked status epilepticus	Potentiating γ and δ subunit-containing GABAergic transmission	[85,86,89,90]
MTL-1	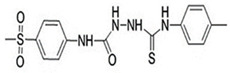	ND	+	ND	+	ND	ND	-	COX-2 inhibitor against neuroinflammation	[92,93,94]
Sebacicacid	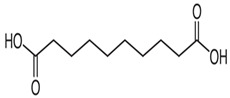	+	+	ND	ND	ND	ND	-	Attenuating overexpression of P-glycoprotein	[97,98]
OV329	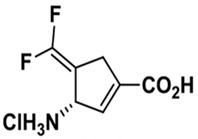	ND	ND	+	ND	ND	ND	i.v. PTZ-induced seizure	Inactivator of GABA aminotransferase	[102,103,104]
KRM-II-81	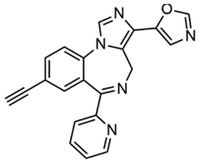	+	+	+	ND	+	+	Kainate-induced chronic epilepsy	Selective modulator of α2/3-containing GABAA receptors	[105,106,107,108,109]

Abbreviations: COX-2 = Cyclooxygenase-2, GABA = γ-Aminobutyric acid, Li-Pilo = Lithium-Pilocarpine, LTG = Lamotrigine, ND = no data, PTZ = pentylenetetrazole, VGSC: voltage-gated sodium channel.

## Data Availability

The data presented in this study are available on request from the corresponding author.

## Abbreviations

ADD	After-discharge duration
ADT	After-discharge threshold
AMPA	α-amino-3-hydroxy-5-methyl-4-isoxazolepropionic acid
ASM	Antiseizure medication
BBB	Blood–brain barrier
COX	Cyclooxygenase
CYP	Cytochrome P450
DRE	Drug-resistant epilepsy
ED50	Median effective dose
ETX	Ethosuximide
GABA	γ-Aminobutyric acid
GABA-AT	GABA aminotransferase
GAERS	Genetic absence epilepsy rat from Strasbourg
GTC	Generalized tonic–clonic
i.p.	Intraperitoneal
i.v.	Intravenous
LCS	Lacosamide
LEV	Levetiracetam
LTG	Lamotrigine
MES	Maximal electroshock seizure
nAChR	Nicotinic acetylcholine receptor
NMDA	N-methyl-D-aspartate
PAM	Positive allosteric modulator
P-gp	P-glycoprotein
PHT	Phenytoin
PTZ	pentylenetetrazole
SA	Sebacic acid
s.c.	Subcutaneous
SE	Status epilepticus
SRS	Spontaneous recurrent seizure
TD50	Median toxic dose
TLE	Temporal lobe epilepsy
VGSC	Voltage-gated sodium channel
VPA	Valproic acid
